# 

**Published:** 1912-04

**Authors:** 


### CONTENTS.

ORIGINAL COMMUNICATIONS:—

The Wassermann Reaction in the Diagnosis of Syphilis. By J. Edgar Paullin, Atlanta, Ga. ........ 1

The Relation of the Eye to General Medicine. By B. Cecil Stockard, M. D., Atlanta, Ga. ........ 11

Breech Presentation—Hydrocephalus—Spina Bifida. By Frederick G. Hodgson, M. D., Atlanta, Ga. ........ 14

EDITORIAL ........ 16

SELECTIONS AND ABSTRACTS ........ 19

